# Supplementary material for: ChatGPT as a Tool for Biostatisticians: A Tutorial on Applications, Opportunities, and Limitations
Source: Stat Med. 2025 Oct 23;44(23-24):e70263. doi: 10.1002/sim.70263 (PMC12548020; doi:10.1002/sim.70263)
Supplement: Supplementary file 1 — Data S1. Supporting Information. [file SIM-44-0-s001.zip › Supplementary Materials Additional.zip!/3.5_Causal_Inferece/3.5_Causal_Chat1.pdf]

# Combining statistical and causal thinking - Clear Memory Request

Please clear memory.

I've cleared the memory of our past conversations. If there's anything you'd like to start fresh with, just let me know!

Take the role of an expert biostatistician for this Chat.

Understood — I'll act as an expert biostatistician for this chat. Feel free to ask anything related to clinical trials, causal inference, survival analysis, longitudinal data, missing data, estimands, or any other biostatistical topic. What would you like to discuss?

I have data from a randomised clinical trial. I want to compare the efficacy of the experimental treatment with that of the control on overall survival in patients with non-small cell lung cancer. In this two-arm RCT, a considerable number of patients in the control arm switched to the experimental treatment after disease progression, while those in the experimental arm never switched.
My goal is to estimate the causal effect of sustained intake of the experimental treatment versus sustained intake of control on Overall survival, had the control group patients not been allowed to switch. Based on the information provided above, please define my target Estimand, following the guidance from the Estimand framework (ICH-E9 addendum).

Based on your setting — a two-arm randomised controlled trial (RCT) in non-small cell lung cancer (NSCLC) with **treatment switching in the control arm after disease progression**, and **no switching in the experimental arm** — your goal is to estimate a **causal treatment effect** under a **hypothetical strategy**. Below is your target estimand defined using the **ICH E9 (R1) Estimand Framework**:

---

## Target Estimand Definition (per ICH E9 (R1))

### 1. Treatment:

- *Intervention:* Sustained use of the experimental treatment as randomised.
- *Comparator:* Sustained use of control treatment as randomised, **without switching** to the experimental treatment after progression (i.e. treatment switching is not permitted in the estimand).

**2. Population:**

- All randomised patients with non-small cell lung cancer, consistent with the intention-to-treat (ITT) principle.
- This includes all eligible patients randomised to either the experimental or control arm, irrespective of subsequent adherence or intercurrent events.

**3. Variable (Endpoint):**

- Overall Survival (OS), defined as time from randomisation to death from any cause.
- Patients still alive at the time of analysis are censored at their last known follow-up date.

**4. Intercurrent Event(s) and Handling Strategy:**

- *Treatment Switching in the Control Arm:*
  - **Handled using a hypothetical strategy** — we ask what the outcome would have been *had the control group not switched* to the experimental treatment after progression.
  - This implies using **causal inference methods** such as inverse probability of censoring weighting (IPCW), structural nested models, or G-estimation to estimate outcomes under the no-switching scenario.
- *Other Intercurrent Events (optional depending on scope):*
  - Events such as loss to follow-up or withdrawal may also need to be addressed (e.g. using hypothetical or treatment policy strategies), depending on their expected impact.

**5. Summary Measure:**

- The **contrast** of interest is the **difference in survival curves**, **hazard ratio**, or **restricted mean survival time (RMST)** comparing:
  - Experimental group under sustained experimental treatment, vs.
  - Control group **had they remained on control treatment** throughout.

---

## Interpretation

This estimand targets the **causal effect of sustained treatment** with the experimental therapy versus sustained control therapy, in a scenario where **control patients did not switch**. It aims to reflect the effect that would have been observed had treatment switching been prohibited and everyone adhered to their initially assigned treatment.

This is **not** a de facto treatment policy estimand (which would allow switching), but a **hypothetical estimand** requiring appropriate adjustment techniques due to post-randomisation deviations (switching).

---

Let me know if you'd like help expressing this in a protocol-ready or publication-ready format, or tailoring the intercurrent event handling for regulatory clarity.

The switching behaviour is affected by a set of different baseline and time-varying covariates. Here, I want to assume that the time-varying confounders that influence the switching behaviour and Overall survival are also themselves affected by prior treatment intake.
For e.g. ECOG score might not only affect whether patients should switch treatment or not, but its value might also be affected by prior treatment intake reflected by the switching behaviour.
In this case, the analytical problem becomes more complex. Can you explain?

Yes, your setting introduces a **time-varying confounding problem with treatment–confounder feedback**, which complicates standard approaches for estimating causal effects. Let's unpack the issues:

---

## 1. Nature of the Problem

You aim to estimate the **causal effect of sustained treatment** (experimental vs control) on **overall survival**, under a **hypothetical no-switching scenario**. However, the challenge is that:

- **Switching behaviour** (an intercurrent event) is influenced by **both baseline and time-varying covariates**.
- These **time-varying covariates**, such as ECOG score or disease progression, **also predict overall survival**.
- Crucially, these covariates are **themselves affected by prior treatment intake**.

This setup creates a **treatment–confounder feedback loop**.

---

## 2. Why Standard Methods Fail

Standard approaches like:

- **Cox regression adjusted for time-varying covariates**, or
- **Naive inverse probability of censoring weighting (IPCW)** that only adjusts for baseline covariates

... will generally **fail to recover the causal effect**, because:

- Time-varying covariates (e.g. ECOG) are **on the causal pathway** from treatment to outcome.
- Simply adjusting for them would **block part of the treatment effect** (introducing **collider bias** or **overadjustment**).
- Not adjusting for them introduces **confounding** (since they influence both switching and outcome).

This is a classical setting described by Robins (1986, 1992) where **time-varying confounders are affected by prior treatment**.

---

## 3. Appropriate Analytical Framework: G-methods

To handle this, you need **causal inference methods** that correctly adjust for **time-varying confounding** in the presence of **treatment-confounder feedback**:

**a. Inverse Probability of Censoring Weighting (IPCW)**

- Censor patients at switching time.
- Estimate the probability of remaining uncensored (i.e., not switching), conditional on **past treatment and covariate history**.
- Use these probabilities to **reweight** observed outcomes, mimicking a pseudo-population where switching does not occur.
- Must include time-varying covariates **as they evolve**, and account for the fact that these are **affected by past treatment**.

**b. Marginal Structural Models (MSMs) with IPTW**

- Model survival as a function of treatment history (e.g. sustained experimental vs control).
- Use **inverse probability of treatment weights** (IPTWs) to account for **time-varying confounding**, incorporating both baseline and time-updated covariates.

**c. G-estimation of Structural Nested Models (SNMs)**

- Estimate the causal effect of treatment by modelling counterfactual outcomes under hypothetical sustained treatment regimens, accounting for the influence of time-varying covariates.

---

## 4. Conceptual DAG Illustration

Here's a simplified causal DAG showing the problem:

```markdown
      L0         L1         L2
       \        / \        /
        \      /   \      /
        A0 → A1 →  A2    ...  (Treatment / Switching)
            \    /  \
             \  /    \
              Y (Overall Survival)
```

Where:

- `L0, L1, L2` = time-varying confounders (e.g. ECOG, tumour burden)
- `A0, A1, A2` = treatment at time t (initial or switched)
- `Y` = outcome (OS)

Arrows from `A` to `L` show **treatment affecting future confounders**, and arrows from `L` to `A` and `Y` show **confounding**.

---

## 5. Implications

- You cannot use **standard regression** to control for post-baseline covariates.

- You must model the **longitudinal treatment and covariate process**, often requiring **sequential models** or **weighted estimation**.
- Correct model specification is crucial — sensitivity analyses are often needed to assess robustness to violations (e.g. unmeasured confounding, model misspecification).

---

Let me know if you'd like help specifying a concrete analysis plan (e.g. IPCW with flexible censoring weights, or MSM implementation using pooled logistic regression), or a schematic DAG to communicate this visually.

I would like to apply the inverse probability of censoring weighting (IPCW) approach to address the bias introduced by switching following the methodology discussed in the following paper: Robins JM, Finkelstein DM. Correcting for noncompliance and dependent censoring in an AIDS clinical trial with inverse probability of censoring weighted (IPCW) log-rank tests. Biometrics. 2000;56(3):779–88.
Briefly summarise the key points from this paper.

Certainly. The 2000 paper by **Robins and Finkelstein**, titled *"Correcting for noncompliance and dependent censoring in an AIDS clinical trial with inverse probability of censoring weighted (IPCW) log-rank tests"*, is a foundational methodological paper that introduced IPCW methods for survival analysis in the presence of **noncompliance and dependent (informative) censoring**. Here's a summary of the key points:

---

## Key Concepts and Contributions

### 1. Problem Setting

- The paper addresses **noncompliance** in clinical trials, particularly **treatment switching** and **dropout**, which lead to **dependent (informative) censoring**.
- Standard survival analysis methods like the **log-rank test** and **Cox regression** assume **non-informative censoring**, which is violated when censoring (e.g. due to switching) depends on **time-varying covariates** or **past treatment history**.

### 2. Proposed Solution: IPCW

- Robins and Finkelstein propose **IPCW estimators** that reweight observed data to account for dependent censoring.
- The key idea:
  - Treat switching as a **censoring event**.
  - Estimate the **probability of remaining uncensored (i.e., not switching)** over time, conditional on the full observed **covariate and treatment history**.
  - Use the **inverse of these probabilities as weights** in a **weighted log-rank test** or **weighted Kaplan–Meier/Cox model**.

### 3. Construction of Weights

- The **censoring model** is fitted using discrete-time logistic regression (or Cox) for the hazard of censoring at each time point, conditional on:
  - **Baseline covariates**
  - **Time-varying covariates**
  - **Past treatment history**
- The IPC weights are calculated as the **inverse of the predicted probability of not being censored up to time t**.

**4. Application and Illustration**

- The method is applied to data from an **AIDS clinical trial**, where patients could **switch** from placebo to active treatment based on intermediate outcomes.
- The IPCW log-rank test provided an estimate of the treatment effect **had switching not occurred**, thus aligning with a **hypothetical estimand**.

**5. Assumptions**

- **No unmeasured confounding**: All variables that jointly affect the censoring (switching) and the outcome are measured and correctly modelled.
- **Positivity**: All patients have a positive probability of remaining uncensored at all time points, conditional on their history.
- **Correct model specification** for the censoring mechanism.

**6. Advantages**

- Allows for unbiased estimation of survival curves or treatment effects under a **hypothetical scenario** (no switching), even when censoring is **informative** and **time-dependent**.
- Extends the standard log-rank and survival analysis tools to more realistic trial settings involving noncompliance.

**7. Limitations**

- Sensitive to **model misspecification** and **violation of the no unmeasured confounding assumption**.
- Requires sufficient overlap in covariate distributions (positivity).
- Can result in **unstable weights** if probabilities of remaining uncensored are close to zero (requires careful diagnostics and possibly weight truncation).

---

## Relevance to Your Study

In your trial, control-arm patients switch to the experimental treatment based on disease progression and other time-varying covariates (e.g. ECOG score). Since these covariates also affect survival and are affected by past treatment, standard methods would be biased.

The **Robins & Finkelstein IPCW method** offers a principled way to estimate the **causal effect of sustained control vs sustained experimental treatment** by:

- Censoring at the time of switching,
- Modelling the switching mechanism using measured covariates and treatment history,
- Reweighting the uncensored patients to reconstruct the counterfactual no-switching scenario.

---

Let me know if you'd like help implementing IPCW in practice (e.g. in R or Stata), choosing covariates, checking model assumptions, or presenting the method clearly in a paper or SAP.

Could you elaborate more on the research question Robins and Finkelstein were trying to answer in their paper, mentioning the comparator arms, possible intercurrent events, outcome definition and population under study.

Certainly. The **Robins and Finkelstein (2000)** paper focused on a methodological challenge arising from **noncompliance and treatment switching** in an AIDS clinical trial. Here's a structured breakdown of the research question they were addressing, framed similarly to the estimand framework for clarity:

---

## 1. Trial Context and Research Question

The authors used data from the **AIDS Clinical Trials Group Protocol 019 (ACTG 019)**, a randomised controlled trial evaluating **zidovudine (AZT)**, an antiretroviral therapy, in asymptomatic HIV-infected individuals.

**Treatment Arms (Intervention/Comparator):**

- **Experimental arm**: AZT (zidovudine) at the outset of the trial.
- **Control arm**: Placebo.

**Objective of the Trial:**

To estimate the effect of **early initiation of AZT** versus **delayed initiation (placebo until disease progression)** on **survival or AIDS-free survival** in asymptomatic HIV-positive individuals.

---

## 2. Intercurrent Events (Post-randomisation Events):

**a. Treatment Switching (Noncompliance):**

- A major intercurrent event in this trial was **treatment switching**: many patients in the **placebo arm** switched to **AZT** after experiencing a significant drop in CD4 count (a marker of immune function).
- This switch was **not random**, but **based on disease progression**, which is **associated with worse outcomes**.
- Patients in the **AZT arm** generally continued on treatment as planned.

**b. Dropout / Loss to Follow-up:**

- Another intercurrent event was **loss to follow-up**, which could be related to disease progression or side effects.

---

### 3. Outcome of Interest:

- The primary outcome was **AIDS-free survival** or **overall survival**.
  - In their illustrative application, Robins and Finkelstein focus on **time to AIDS or death** (a composite endpoint).
- Patients were followed from **randomisation** until:
  - Onset of AIDS or death (event), or
  - Loss to follow-up or administrative censoring (censored).

---

### 4. Population Under Study:

- The analysis included all **randomised participants**, in line with the **intention-to-treat (ITT) principle**.
- However, the **estimand** of interest diverged from ITT: it aimed to estimate the effect **had all participants remained on their originally assigned treatment throughout follow-up** — i.e. a **hypothetical estimand**.

---

### 5. Target Estimand / Research Question:

**What is the causal effect of being continuously treated with AZT versus never receiving AZT on AIDS-free survival**, in the population of HIV-positive individuals enrolled in ACTG 019, **under a hypothetical scenario where patients did not switch treatments**?

This differs from the standard ITT estimand, which would include the real-world effect of switching.

---

### Why IPCW Was Needed:

- Because **switching was strongly dependent on CD4 count**, a **time-varying confounder affected by prior treatment**, the analysis required methods that could:
  - Handle **informative censoring** (i.e. switching related to prognosis),
  - Avoid **bias from standard covariate adjustment** (which could block part of the treatment effect or induce collider bias),
  - Allow estimation of **counterfactual outcomes** under no-switching scenarios.

Hence, Robins and Finkelstein proposed using **IPCW log-rank tests** and **IPCW-weighted survival curves** to estimate what survival would have looked like **if switching had not occurred**.

---

Let me know if you'd like this rephrased in a more formal statistical analysis plan style, or if you want a schematic DAG of this setting.

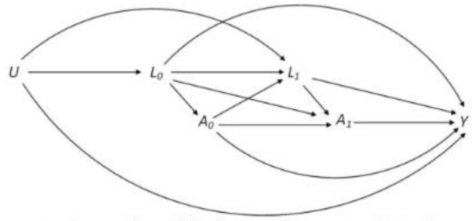


**Fig. 1** Graphical representations of our research focus and the bias of naive methods for time-varying confounding in the presence of treatment-confounder feedback: This Directed acyclic graph (DAG) represents the post-progression period for patients in the control arm. Following disease progression, the decision as to whether or not a patient in the control group continues to take its treatment ($A_0$) or ($A_1$) depends on predictive factors measured at or before the time of disease progression ($L_0$). Again, we assume that treatment intake ($A_0$) affects outcome (Y) and influences a set of TVCs at a later time-point ($L_1$). These covariates are also assumed to impact the clinician's decision as to whether or not a patient should subsequently change treatments ($A_1$) introducing time-varying confounding and treatment-confounder feedback through $L_1$

Attached, I provide you with a directed acyclic graph (DAG) that represents our research problem.
To simplify this DAG and take into consideration this deterministic information provided below, I restrict my DAG to the post-progression period for patients in the control arm only and consider two follow-up time points, 1 & 2.
Here A(k) is deterministically 0 if A(k-1)=0 AND L(k-1) (reflecting progression status)=0 (no progression).
Following disease progression, the decision as to whether or not a patient in the control group continues to take their treatment (A0 or A1) depends on predictive factors measured at or before the time of disease progression (L0).
Again, we assume that treatment intake (A0) affects outcome (Y) and influences a set of TVCS at a later time-point (L1).
These covariates are also assumed to impact the clinician's decision as to whether or not a patient should subsequently change treatments (A1), introducing time-varying confounding and treatment confounder feedback through L1.
I will emphasise again that in our case scenario, patients in the control group do not switch unless they progress, and all patients in the experimental group never switch.
I still want to estimate in my outcome model the causal effects of sustained treatment interventions (always taking control versus always taking experimental) under a hypothetical strategy where control patients do not switch treatment.
I want you to use this DAG as a basis for performing the appropriate IPCW analyses and provide me with the analysis R-script.
You may assume that the dataset is in long format, with variables: id, time (in months and increases with increments of 1), death indicator, switching indicator, baseline covariates
repeated over time points, time-varying covariates at each time pt.

**Uploaded DAG: Post-Progression Control Arm Structure**

Uploaded DAG: Post-Progression Control Arm Structure

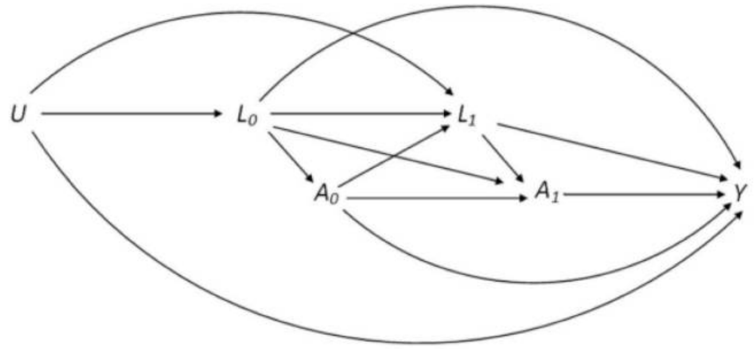


**Fig. 1** Graphical representations of our research focus and the bias of naive methods for time-varying confounding in the presence of treatment-confounder feedback: This Directed acyclic graph (DAG) represents the post-progression period for patients in the control arm. Following disease progression, the decision as to whether or not a patient in the control group continues to take its treatment ($A_0$) or ($A_1$) depends on predictive factors measured at or before the time of disease progression ($L_0$). Again, we assume that treatment intake ($A_0$) affects outcome ($Y$) and influences a set of TVCs at a later time-point ($L_1$). These covariates are also assumed to impact the clinician's decision as to whether or not a patient should subsequently change treatments ($A_1$) introducing time-varying confounding and treatment-confounder feedback through $L_1$

Thank you — this DAG clearly illustrates **treatment-confounder feedback** in the control arm post-progression. Based on this structure and your goal to estimate a **hypothetical estimand** under **no switching**, an **IPCW approach** is appropriate.

Below is an **R analysis template** for applying **IPCW** to your data. It assumes:

- Long-format data (`id`, `time`, `death`, `switch`, baseline covariates, time-varying covariates).
- Patients in the control arm may switch after progression, governed by past covariates.
- The treatment switching variable (`switch`) is treated as **informative censoring**.

---

## 🔧 R Script for IPCW Estimation (Sustained Treatment Effect)

```r
# Load libraries
library(survival)
library(dplyr)
library(splines)
library(ipw)

# Step 1: Fit the model for censoring (switching)
# This is the IPCW part. Estimate Pr(not switched | covariate history)
data <- data %>%
  arrange(id, time) %>%
  group_by(id) %>%
  mutate(
    switched = ifelse(switch == 1, 1, 0),  # censoring indicator
    death_event = ifelse(death == 1, 1, 0)
  )

# Logistic regression model for staying uncensored (i.e., not switched)
# Include baseline and time-varying covariates
censor_model <- glm(switched ~ time + tvcov1 + tvcov2 + baseline1 + baseline2,
                    family = binomial(), data = data)

# Predict the probability of *not switching* at each time
data$psurv_uncensored <- 1 - predict(censor_model, type = "response")

# Step 2: Compute stabilized IPC weights
data <- data %>%
  group_by(id) %>%
  mutate(
```

```
    weight_numer = cumprod(rep(1, n())),  # numerator = 1 for all (or include minimal model)
    weight_denom = cumprod(psurv_uncensored),
    ipcw = weight_numer / weight_denom
  )

# Step 3: Fit a weighted Cox model for the effect of randomised treatment
# We assume a hypothetical strategy: censor at switching
# Restrict to pre-switching time or use IPCW weights
cox_ipcw <- coxph(Surv(time, death_event) ~ treatment,
                  data = data,
                  weights = ipcw,
                  robust = TRUE)

summary(cox_ipcw)
```

---

## 🔍 Key Variables Expected

- `` `treatment` ``: indicator for randomised treatment (1 = experimental, 0 = control).
- `` `switch` ``: indicator (1 = switched at this time, 0 = not switched).
- `` `death` ``: indicator (1 = died at this time, 0 = alive).
- `` `tvcov1` ``, `` `tvcov2` ``: time-varying covariates (e.g. ECOG, progression status).
- `` `baseline1` ``, `` `baseline2` ``: baseline covariates.

---

## ✅ Assumptions

- **No unmeasured confounders** for the switching process (all predictors of switching and outcome are included).
- **Correct model specification** for the switching model.
- **Positivity**: All patients have non-zero probability of remaining uncensored (i.e. not switching) given their history.

---

Let me know if you'd like to:

- Incorporate **truncation** of extreme weights,
- Adapt this for **time-dependent Cox models**,
- Visualise **IPCW-adjusted survival curves** (e.g. using `` `survey::svykm` ``),
- Extend to **RMST estimation** or **bootstrap** standard errors.

I'm also happy to tailor the model terms if you give me the exact covariates from your data.
